# Impact of pulmonary artery flow distribution on Fontan hemodynamics and flow energetics

**DOI:** 10.1007/s00247-023-05591-z

**Published:** 2023-03-07

**Authors:** Elizabeth K. Weiss, Joshua D. Robinson, Aparna Sodhi, Michael Markl, Cynthia K. Rigsby

**Affiliations:** 1 Department of Radiology, Feinberg School of Medicine, Northwestern University, 737 North Michigan Avenue Suite 1600, Chicago, IL 60611 USA; 2 Department of Pediatric Cardiology, Ann & Robert H. Lurie Children’s Hospital, Chicago, IL USA; 3 Department of Medical Imaging, Ann & Robert H. Lurie Children’s Hospital, Chicago, IL USA

**Keywords:** Fontan, 4D flow MRI, Viscous energy loss, Flow distribution, Cardiac MRI

## Abstract

**Background:**

With improved life expectancy following Fontan palliation, there is an increasing population of patients with a total cavopulmonary connection. However, there is a poor understanding of which patients will experience Fontan failure and when. 4D flow MRI has identified several metrics of clinical interest, but longitudinal studies investigating hemodynamics in Fontan patients are lacking.

**Objective:**

We aimed to investigate the relationship between flow distribution to the pulmonary arteries and regional hemodynamic metrics in a unique cohort with follow-up 4D flow MRI.

**Materials and methods:**

Patients with > 6 months of 4D flow MRI follow-up were included. Flow distribution from the caval veins to pulmonary arteries was measured in addition to regional measures of peak velocity, viscous energy loss (EL_mean_ and EL_tot_), and kinetic energy.

**Results:**

Ten patients with total cavopulmonary connection (17.7 ± 8.8 years at baseline, follow-up: 4.4 ± 2.6 years) were included. Five subjects had unequal flow distribution from the IVC to the pulmonary arteries at baseline. Over time, these subjects tended to have larger increases in peak velocity (39.2% vs 6.6%), EL_mean_ (11.6% vs -38.3%), EL_tot_ (9.5% vs -36.2%), and kinetic energy (96.1% vs 36.3%) in the IVC. However, these differences were statistically insignificant. We found that changes in EL_mean_ and EL_tot_ were significantly associated with changes in peak velocity in the caval veins (R^2^ > 0.5, *P* < 0.001).

**Conclusion:**

Unequal flow distribution from the IVC may drive increasing peak velocities and viscous energy losses, which have been associated with worse clinical outcomes. Changes in peak velocity may serve as a surrogate measure for changes in viscous energy loss.

## Introduction

Single ventricle physiology is among the most severe forms of CHD. These patients undergo palliative vascular surgical procedures to achieve the Fontan circulation in which systemic venous return is routed directly to the lungs (total cavopulmonary connection). This procedure creates a circulation with chronically elevated venous pressure, without mixing of venous return prior to pulmonary perfusion and without pulsatile blood flow within the pulmonary arteries. These unique hemodynamic characteristics are thought to mediate various adverse outcomes, such as hepatic fibrosis [1] and pulmonary arteriovenous malformations [2, 3].

While life expectancy among Fontan patients has increased in the previous decades, 70% of patients experience signs of Fontan failure by the age of 50 [4, 5]. Despite regular, imaging-based follow-up, clinicians are unable to reliably identify early signs of Fontan failure for early intervention, and biomarkers to tailor patient management are lacking. There has been significant effort to address this knowledge gap. Clinical studies have identified several cardiac MR metrics that correlate with Fontan outcomes including ejection fraction [6], end diastolic volume [7], and aortopulmonary collateral burden [8]. Given the unique hemodynamics of the Fontan circulation, additional studies have leveraged the potential of 4D flow MRI to measure Fontan 3D blood flow dynamics in-vivo to derive new metrics for the assessment of Fontan function, such as flow distribution from the caval veins to the pulmonary arteries [9]. Flow distribution from the inferior vena cava, which carries hepatic factor to the lungs, has been hypothesized to play a role in the formation of pulmonary venous-arterial malformations [10]. Additionally, 4D flow MRI studies have investigated changes in flow energetics, demonstrating that ventricular kinetic energy, a measure of total energy due to blood flow, is significantly decreased in Fontan patients [11, 12] and that viscous energy loss, a measure of energy lost due to flow inefficiencies, correlates with decreased exercise capacity [13, 14].

While there has been substantial effort to characterize Fontan hemodynamics, there is a dearth of follow-up studies to characterize longitudinal changes in Fontan hemodynamics. To begin addressing this gap, we aimed to analyze a cohort of Fontan patients with at least two 4D flow scans to quantify longitudinal changes in Fontan hemodynamics biomarkers, such as viscous energy loss and peak velocity, and their relationship with pulmonary artery flow distribution.

## Methods

### Study cohort

This study utilized a retrospective database consisting of 1041 patients receiving 4D flow MRI during clinically indicated cardiovascular MR studies at Lurie Children’s Hospital between May 2012 and January 2022. Subjects were included if they were status-post Fontan procedure with at least two valid 4D flow scans separated by at least 6 months (*n* = 12). Those who did not have a lateral tunnel Fontan or extracardiac Fontan procedure at both scans were excluded (1 patient). Further, scans containing artifact from medical devices (e.g., coils, stents) that prevented evaluation of all 4 vessels of the total cavopulmonary connection were excluded (1 patient). In patients with more than two valid 4D flow scans, the earliest acquisition with cardiac function measurements served as baseline and the most recent as the follow-up exam. Ejection fraction and end diastolic volume indexed to body surface area were collected for the dominant ventricle at baseline and follow-up from the MR radiologist report as measured by CINE imaging. This health insurance portability and accountability act (HIPAA)-compliant study was approved by the institutional review board (IRB) at Lurie Children’s Hospital, and patients were retrospectively enrolled with a waiver of consent.

### MR imaging

All exams were performed on 1.5 T MRI systems (Aera, Siemens Healthcare, Erlangen, Germany). Each patient underwent standard-of-care cardiothoracic MRI, including gadolinium (Ablavar, Lantheus Medical Imaging, North Billerica, MA, or Magnevist or Gadavist, Bayer HealthCare, LLC, Pittsburgh, PA) or ferumoxytol (Feraheme, AMAG Pharmaceuticals, Waltham, MA) enhanced MR angiogram and CINE imaging followed by 4D flow MRI with whole heart coverage (respiratory navigator gating, EKG gating—7 retrospectively gated scans and 13 prospectively gated scans). 4D flow MRI scan parameters were as follows: spatial resolution = 1.95–4.67 mm × 1.43–2.5 mm × 1.4–2.9 mm, FOV = 250 × 173–370 × 278 mm, TR = 9.6–11.2 ms (temporal resolution = 4*TR), TE = 2.36–2.98 ms, flip angle = 15–25°, and velocity encode (venc) = 80–150 cm/s.

### 4D flow data analysis – preprocessing and 3D total cavopulmonary connection segmentation

Each 4D flow scan was manually preprocessed, including corrections for phase offset errors (Maxwell terms, eddy currents), noise masking, and velocity anti-aliasing using an in-house tool programmed in Matlab (MathWorks, Natick, MA). Similar to previously reported strategies [15], a mean sum square phase contrast MR angiogram was calculated and used for manual 3D segmentation of the total cavopulmonary connection (Mimics, Materialize, Leuven, Belgium). Segmentation terminated at the hepatic veins in the IVC/baffle region, the first branch of the SVC, and at the segmental branches in the pulmonary arteries. For flow distribution analysis, the IVC and SVC were manually segmented from the whole total cavopulmonary connection segmentation.

### 4D flow data analysis – caval vein blood flow distribution

**Fig. 1:**
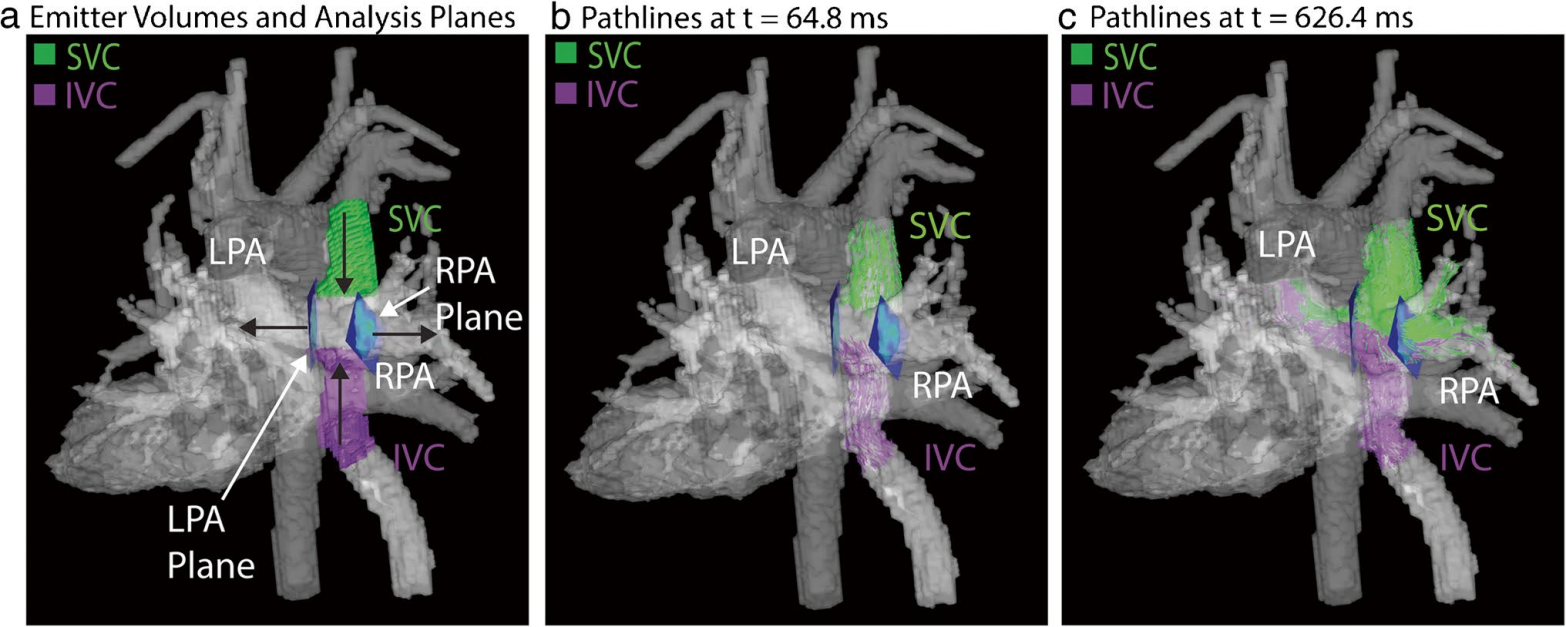
Flow distribution measurement. The IVC and SVC emitter volumes (purple and green, respectively) as well as manually placed analysis planes are shown in (**a**). Black arrows indicate the direction of flow within the total cavopulmonary connection. Pathlines at two progressive time points are shown in (**b**)–(**c**). Patient shown was 16 year-old male, status post extracardiac Fontan. Native anatomy included double outlet right ventricle and pulmonary valve atresia. Images depict segmentations derived from a phase contrast angiogram calculated from 4D flow MRI

The flow distribution from the IVC and SVC to the right and left pulmonary arteries was measured in each scan using a previously validated method [16] (Mimics Materialize, Leuven, Belgium). The segmented IVC and SVC were used as flow emitter volumes, and 2D analysis planes were manually placed at the entrance to the LPA and RPA (Fig. 1a). Pathlines, defined as the calculated path of a particle due to the velocity vector fields, were then emitted with a density of 30 emitters/cm^3^ from both the IVC and SVC volumes (Fig. 1b–c). The pathlines and plane locations were exported, and the number of pathlines entering the LPA and RPA from each caval vein was counted using an in-house tool developed in Matlab. The flow distribution was calculated as follows for both the SVC and IVC:

1 $$Flow\;Distribution\;to\;LPA=\frac{pathlines\;reaching\;LPA\;plane}{pathlines\;reaching\;LPA+pathlines\;reaching\;RPA}$$

2 $$Flow\;Distribution\;to\;RPA=\frac{pathlines\;reaching\;RPA\;plane}{pathlines\;reaching\;LPA+pathlines\;reaching\;RPA}$$

To isolate differences due to moderate-extremely unequal flow, unequal flow distribution was defined as < 30% or > 70% flow distribution to the LPA.

### 4D flow data analysis – voxel-wise hemodynamic maps

**Fig. 2:**
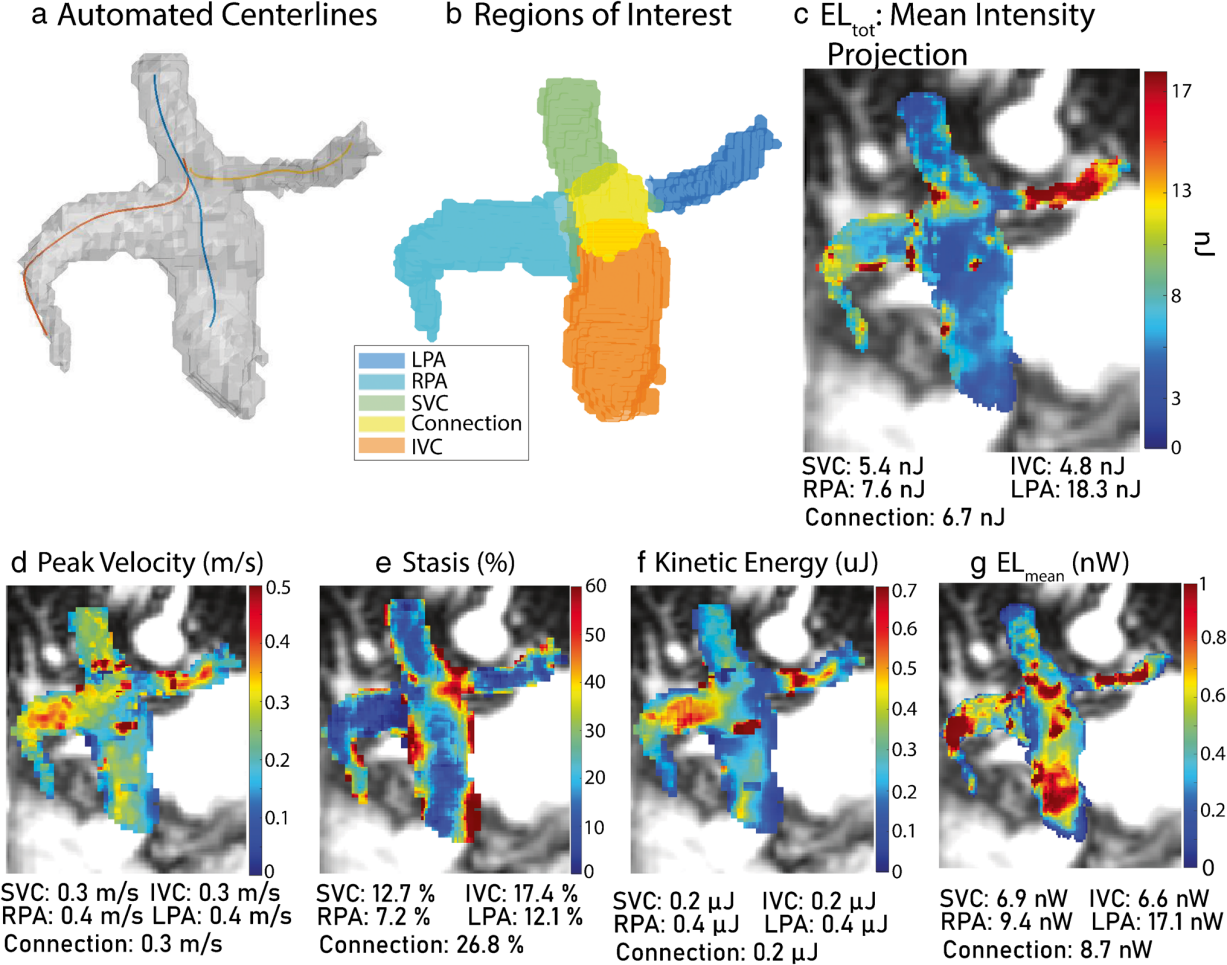
Voxel-wise analysis pipeline. Centerlines are automatically calculated (**a**), and planes are placed to denote the regions of interest (**b**). Example quantitative maps for all metrics are shown in (**c**)–(**g**). Patient shown was 20-year-old male, status-post lateral tunnel Fontan. Native anatomy included double outlet right ventricle and mitral atresia. Coronal magnitude images from 4D flow MRI are shown. EL, energy loss

Based on the 3D total cavopulmonary connection segmentation, vessel centerlines were automatically calculated for the RPA, LPA, and caval veins + connection region (Fig. 2a). 2D analysis planes were placed along the centerlines to delineate the boundaries of the 3D regions of interest (ROI, Fig. 2b). To accommodate variance in cardiac gating (prospective vs retrospective), the percent of cardiac cycle captured by each 4D flow MRI scan was calculated. The minimum was selected, and all 4D flow MRI data were truncated to include the same fraction of the cardiac cycle. All data was interpolated to 1mm^3^ isotropic spatial resolution, and 5 hemodynamic metrics (summarized in Table 1) were calculated. Peak velocity (Fig. 2d) at each voxel was measured, and the 98th percentile was reported for each ROI. Stasis (Fig. 2e), defined as percent of time frames with velocity < 0.1 m/s, was measured per voxel and averaged over each ROI. Kinetic energy (KE, Fig. 2f) was calculated using Eq. 3 at each voxel and summed over time. The mean in each ROI was reported. Viscous energy dissipation (ϕ_v_) was calculated at each voxel over time using Eq. 4 and multiplied by voxel volume and blood viscosity (μ) to measure the voxel-wise energy loss (EL) rate. This was integrated over time on a voxel-wise basis to measure total energy loss (EL_tot_, Fig. 2c), and the mean over each ROI was reported. Energy loss was also averaged over time on a voxel-wise bases to measure mean energy loss rate (EL_mean_, Fig. 2g) and averaged again over each ROI. For all measurements, blood was assumed Newtonian and incompressible with a density (ρ) of 1060 kg/m^3^ and viscosity (μ) of 3.2 cP.

**Table 1:** Summary of voxel-wise hemodynamic metrics measured

Metric	Voxel-wise calculation	Regional measurement
Peak velocity (m/s)	Max velocity over all time points	98th percentile of peak velocity in ROI
Stasis %	% time points with velocity < 0.1 m/s	Mean over ROI
KE (J)	Kinetic energy (EQ 3) measured at each voxel and summed over time	Mean over ROI
EL_tot_ (J)	Viscous dissipation (EQ 4) measured at each voxel, multiplied by voxel size and viscosity, and integrated over time	Mean over ROI
EL_mean_ (W)	Viscous dissipation (EQ 4) measured at each voxel multiplied by voxel size and viscosity, averaged over time	Mean over ROI

*KE* kinetic energy, *EL* energy loss, *ROI* region of interest

3 $$KE=\frac12\rho\ast voxel\;volume\ast(velocity\;magnitude)^2$$

4 $$\begin{array}{l}\phi_v=\frac12\sum_i\sum_j\left[\left(\frac{\partial v_j}{\partial x_i}+\frac{\partial v_i}{\partial x_j}\right)-\frac23(\nabla\bullet V)\delta_{ij}\right]^2\\{Where:\delta}_{ij}=1\;for\;i=j\\\delta_{ij}=0\;for\;i\neq j\end{array}$$

### Statistical analysis

The change (Δ) of each parameter was calculated as the difference between follow-up and baseline, normalized to the follow up time in years. Wilcoxon Rank sum tests (α = 0.05) were used to compare patient groups with unequal vs equal flow distribution from the IVC and to compare patient groups with lateral tunnel Fontan to extracardiac Fontan. Sign rank tests were used to assess for significant changes from baseline to follow-up. Association between hemodynamics metrics were assessed with Spearman correlation followed by linear regression. Hemodynamic statistics are reported as median ± interquartile range. A post-hoc power calculation was completed for all correlations, comparisons between groups with unequal or equal flow distribution form the IVC, and sign rank tests assessing stability of regional hemodynamic metrics over the follow-up.

## Results

### Study cohort

Ten patients were included in the study cohort (17.7 + 8.8 years old at baseline, four female, Table 2) with an average follow up time of 4.4 ± 2.6 years. Two patients had general anesthesia during both baseline and follow-up, and two patients had general anesthesia during their baseline scan only. Four patients received gadolinium-based contrast at both scans, one patient received ferumoxytol at both scans, and five patients received gadolinium-based contrast at baseline and ferumoxytol at follow-up. Five patients had an extracardiac Fontan conduit, and the remaining five had a lateral tunnel Fontan. All patients had less than 10% decline in ejection fraction and end diastolic volume indexed to body surface area and were considered clinically stable over their follow-up period (Table 2).

**Table 2:** Description of the cohort demographics, Fontan type, and cardiac function metrics

Subject	Age (gender)	GA (BL/FU)	F/u time (years)	Fontan type	EF (BL/FU)	EDVi (BL/FU)
1	15 (F)	Y/Y	0.83	Extracardiac	50.5/50	122/116
2	21 (M)	N/N	7.1	Lateral tunnel	52/50	57/70
3	13 (M)	Y/Y	2.8	Extracardiac	48/60.2	86/85
4	16 (M)	N/N	3.4	Lateral tunnel	49/49	128/120
5	28 (M)	N/N	2.3	Lateral tunnel	55/49	115/98
6	35 (F)	N/N	5.1	Lateral tunnel	38/42	60/60
7	9 (F)	N/N	3.8	Extracardiac	50/52	65/55
8	22 (M)	N/N	3.3	Lateral tunnel	47/44	138/143
9	9 (M)	Y/N	5.3	Extracardiac	22/20	108/63
10	9 (F)	Y/N	9.9	Extracardiac	46/44	109/84

No patients were considered to have a clinically significant worse EF or EDVi at follow-up compared to baseline. Age is in years

*GA* general anesthesia, *EF* ejection fraction, *EDVi* end diastolic volume indexed to body surface area, *BL* baseline, *FU* follow-up

### Hemodynamic metrics

Overall, there was a significant increase in kinetic energy in the left pulmonary artery, total energy loss in the superior vena cava, and peak velocity and kinetic energy in the inferior vena cava and the connection region between baseline and follow-up MRI (Table 3). There were no significant differences in any hemodynamic metrics in the right pulmonary artery. All other hemodynamic measures were stable over time (*P* > 0.2, Table 3). Additionally, there was no difference in any metric in any region of interest when comparing patients with lateral tunnel Fontan vs extracardiac conduit Fontan (*P* > 0.15).

**Table 3:** Median total change normalized to follow-up duration ± interquartile range for each metric and region of interest

	LPA	RPA	Connection	SVC	IVC
ΔPeak velocity (m/s/year)	0.002 ± 0.03 (0.8)	-0.002 ± 0.03 (0.9)	**0.01 ± 0.01 (0.05)**	-0.0004 ± 0.8 (0.8)	**0.02 ± 0.07 (0.02)**
ΔStasis (%/year)	-0.3 ± 3.6 (0.7)	0.65 ± 4.8 (0.5)	-0.2 ± 4.6 (0.8)	0.3 ± 11.6 (0.6)	-0.1 ± 4.6 (0.8)
ΔKE (μJ/year)	**0.05 ± 0.2 (0.03)**	0.03 ± 0.1 (0.2)	**0.03 ± 0.50(0.05)**	0.02 ± 0.02 (0.4)	**0.02 ± 0.04 (0.04)**
ΔEL_tot_ (nJ/year)	-0.15 ± 3.3 (0.4)	-0.52 ± 4.5 (0.7)	-0.13 ± 68 (0.6)	**-0.16 ± 0.73 (0.04)**	-0.19 ± 1.9 (0.9
ΔEL_mean_ (nW/year)	-0.14 ± 0.9 (0.6)	-0.49 ± 8.0 (0.9)	-0.21 ± 1.9 (0.2)	-0.4 ± 0.9 (0.2)	-0.4 ± 1.7 (0.7)

*P*-values are in parenthesis. Significant changes in flow metrics are indicated by bold type (*P* < 0.05)

*KE* kinetic energy, *EL* energy loss

### Impact of flow distribution on longitudinal changes in Fontan hemodynamics

**Fig. 3:**
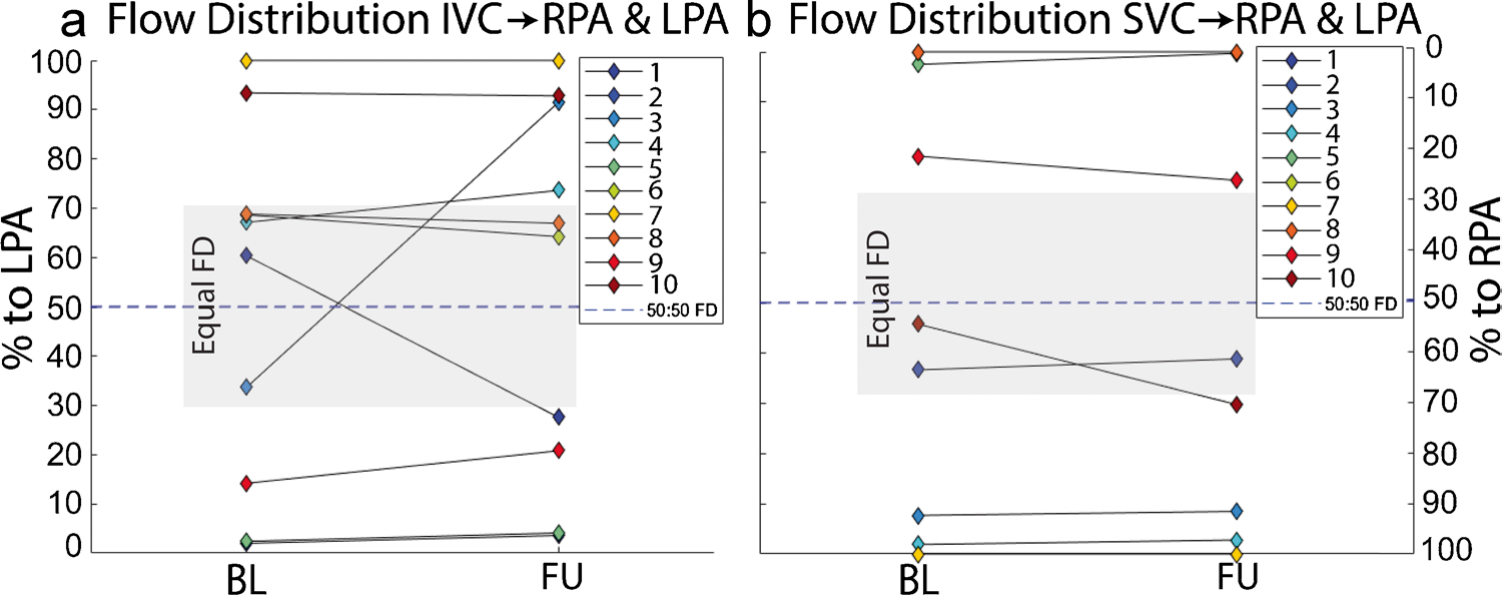
Flow distribution from the IVC (**a**) and SVC (**b**) for all 10 patients at baseline and follow-up. Gray box indicates region of equal flow distribution and dashed line marks exactly equal flow distribution. FD, flow distribution; BL, baseline; FU, follow-up

At baseline, five patients had unequal flow distribution from the IVC to the left and right lungs, while 8 had unequal flow distribution originating from the SVC (Fig. 3). All patients with unequal flow distribution from the IVC and SVC at baseline maintained unequal flow distribution at follow-up. Over the entire cohort, there was no significant change in flow distribution from baseline to follow up (*P* > 0.9 for IVC and SVC). However, two subjects (subjects 1 and 3) with equal flow distribution from the IVC at baseline had substantial changes at follow-up. Subject 1 had a stent placed in the distal Fontan conduit between baseline and follow-up. Subject 3 had increased LPA luminal area and new narrowing at the conduit-IVC anastomosis at follow-up compared to baseline. The pulmonary artery receiving the majority of the flow changed from baseline to follow-up in both subjects. Neither subject had substantial change in flow distribution from the SVC from baseline to follow-up.

**Fig. 4:**
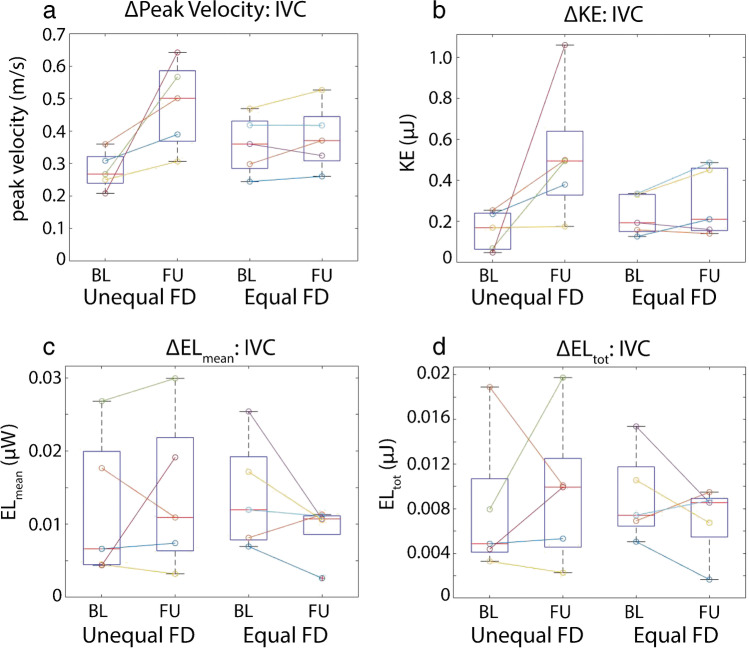
Voxel-wise hemodynamics. ΔPeak velocity (**a**), ΔKinetic energy (**b**), Δmean energy loss (**c**), and Δtotal energy loss (**d**) stratified by baseline flow distribution (FD) from the IVC (unequal vs equal, *n* = 5 in each group). Patients with unequal flow distribution at baseline tended to show larger increases in all 3 metrics in the IVC. FD, flow distribution; BL, baseline; FU, follow-up; EL, energy loss; KE, kinetic energy

The cohort was further stratified by flow distribution from the IVC at baseline (*n* = 5 equal flow distribution, *n* = 5 unequal flow distribution). As shown in Fig. 4, unequal flow distribution from the IVC at baseline was associated with a non-significant increase in peak velocity at follow-up (Δpeak velocity = 0.024 ± 0.066 m/s/year, *P* = 0.06, 39.2% increase) in the IVC, while patents with equal flow distribution at baseline presented with more stable hemodynamics over time (Δpeak velocity = 0.003 ± 0.038 m/s/year, *P* = 0.44, 6.6% increase). Unequal flow distribution at baseline also resulted in higher total energy loss and mean energy loss rate in the IVC at follow up (increase of 9.5% and 11.6%). In contrast, patients with equal flow distribution presented with reduced total energy loss and mean energy loss rate at follow-up (36.2% and 38.3%). Additionally, those with unequal flow distribution at baseline had a larger increase in kinetic energy compared to those with equal flow distribution at baseline (96.1% vs 36.3% increase). This analysis was not repeated in the SVC as only two patients had equal flow distribution at baseline.

### Relationships between metrics of Fontan flow dynamics

**Fig. 5:**
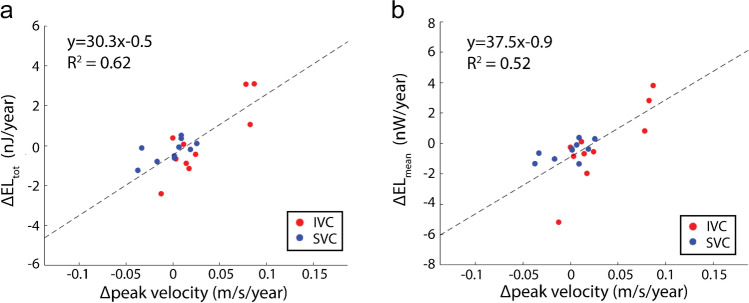
Change in EL and peak velocity in the caval veins. Linear regression relates total changes normalized to follow-up duration in total energy loss with peak velocity (**a**) and changes in mean energy loss with peak velocity (**b**) in the caval veins. Model reported included both SVC and IVC measurements. EL, energy loss

Spearman correlation revealed a significant positive association between Δtotal energy loss and Δpeak velocity in the IVC (rho = 0.66, *P* = 0.04) and SVC (rho = 0.70, *P* = 0.03). There was also a significant association between Δmean energy loss and Δpeak velocity in the IVC (rho = 0.72, *P* = 0.02) but not in the SVC (rho = 0.59, *P* = 0.08). When combining the caval vein measurements, a linear regression model relating changes in energy loss to changes in peak velocity revealed Δpeak velocity as a significant predictor of both increased Δtotal energy loss (R^2^ = 0.62, *P* < 0.001, Fig. 5a) and Δmean energy loss (R^2^ = 0.52, *P* < 0.001, Fig. 5b).

### Post-hoc power analyses

Comparisons of hemodynamic metric changes in those with unequal flow distribution to those with equal flow distribution had the following achieved power: 10% for peak velocity, 25% for kinetic energy, 12% for total energy loss, and 8% for mean energy loss. Sign rank tests assessing total differences in hemodynamic metrics, normalized to follow-up duration, had achieved power of < 30% in all but three comparisons: kinetic energy in SVC (78% power) and peak velocity and kinetic energy in the connection region (78% and 38% power, respectively). Correlations between total energy loss and peak velocity had an achieved power of 77% in the SVC and 68% in the IVC. Correlations between mean energy loss and peak velocity had an achieved power of 53% in the SVC and 82% in the IVC.

## Discussion

The aim of our study was to analyze longitudinal changes in Fontan hemodynamics in a small cohort of Fontan patients with unique multi-year 4D flow MRI follow-up data. We utilized a novel volumetric analysis method with voxel-wise maps of Fontan 3D flow dynamics, including measurement of viscous energy losses, in combination with previously described methods for measuring flow distribution to the pulmonary arteries [9]. Our study had two main findings: (1) unequal flow distribution from the IVC to the pulmonary arteries may be predictive of increased peak velocities and viscous energy losses at follow-up and (2) changes in peak velocities were highly correlated with altered viscous energy losses in the caval veins, suggesting that peak velocities in the SVC and IVC, measures that can be attained with current clinical tools, may serve as a surrogate for changes in viscous energy loss.

Unequal flow distribution from the IVC to the pulmonary arteries has been previously associated with increasing risk of pulmonary arteriovenous malformations (PAVMs) [2, 3, 10]. Our results suggest that the unequal flow distribution may also contribute to worsening flow inefficiencies (increased energy loss). Worsening viscous energy loss has also been shown to be correlated with impaired exercise capacity [13, 14] and increased burden of hepatic fibrosis. Thus, unequal flow distribution from the IVC to the pulmonary arteries may drive adverse outcomes by multiple mechanisms. It may be that geometries predisposed to unequal distribution [9] also predispose to degraded flow efficiency. It is also possible that the asymmetric flow patterns drive inefficient hemodynamics. Further, larger studies should investigate the relationship between IVC flow distribution and viscous energy losses and analyze possible relationships between IVC flow distribution with clinical outcome metrics.

We chose to define equal flow distribution as 30–70% flow to the LPA to assess differences due to moderate-severely unequal distribution. In one computational fluid dynamics study [17], optimal IVC flow distribution was calculated in 5 patients, and 4 of those 5 patients had an optimal flow distribution between 30 and 70% to the LPA. Additionally, a larger study in 100 patients [18] found the 25th percentile of flow distribution from the IVC to the LPA to be 31%. Thus, 30% flow to the LPA was used as the lower limit. We chose a symmetrical range about the ideal 50:50 flow distribution, giving an upper limit of 70% flow to the LPA. Future studies should investigate the impact of equal flow distribution definition. Additionally, it is important to note that pathline calculations are imperfect, with many pathlines terminating prior to reaching either analysis plane in the pulmonary arteries. In our cohort, an average of 15% of pathlines with more than 3 times points reached either plane. This did not significantly vary from baseline to follow-up nor between SVC and IVC measurements.

We also found that changes in peak velocities in both caval veins correlate strongly with changes in total energy loss and mean energy loss rate. This association may be due to worsening conduit-IVC mismatch. It has been previously shown that conduit-IVC mismatch increases mean velocities in the conduit and that a higher degree of mismatch is associated with increased kinetic energy and flow disturbances resulting in increased viscous energy loss [19, 20]. Additionally, since viscous energy loss has been implicated by several studies as being correlated with outcomes in Fontan patients, [11, 13, 14, 21], it is a hemodynamic metric of clinical interest. However, it requires dedicated software to measure. Our findings suggest that changes in peak velocity could serve as a simpler surrogate for changes in viscous energy loss. Larger studies should investigate this relationship using peak velocities measured in a plane or measured by echocardiography, as these methods are more common clinically than the 3D peak velocities measured in this study.

In addition to these two main findings, we reported that kinetic energy increased over follow-up in the LPA and that total energy loss increased in the SVC. The changes in the LPA may reflect increased total blood flow, due to increasing body size with age, without increase in lumen area, causing increased mean velocities, and thus increased kinetic energy. Additionally, a recent CMR study [22] found that, over time, the SVC cross-sectional area decreases. This altered geometry may contribute to increased flow inefficiencies and thus increased total energy losses.

### Limitations

The most substantial limitation to our study was the small cohort size, limiting our power to detect statistically significant differences. However, patients with single ventricle physiology who have completed the Fontan connection are rare (1062 operations per year in the USA) [23], and our study cohort is unique in that it includes multi-year 4D flow MRI data. While the differences observed in energy loss due to IVC flow distribution are not significant, our results suggest that IVC flow distribution may be an important metric in future, larger, longitudinal studies assessing hemodynamics and clinical outcomes. Additionally, since the cohort was clinically stable over the 4.4 year mean follow-up duration, we were unable to associate hemodynamics metrics with cardiac function measures. However, energy loss and kinetic energy, which our data suggests may increase more in patients with unequal flow distribution from the IVC, have been shown to correlate with clinical outcomes [14, 19, 21]. Given the small sample size, we were unable to systematically investigate the impact of scan parameters such as spatial and temporal resolution. However, previous studies have shown that flow distribution is robust to changes in spatial resolution [16]. Additionally, our voxel-wise mapping method aimed to reduce the impact of differences in spatial resolution through 1 mm^3^ isotropic interpolation. Reduced acquired resolution may increase partial volume effects, leading to smoothed hemodynamic metric maps. This likely has limited impact on averaged measures (such as kinetic energy) but may reduce the measured peak velocities. Additionally, variability in signal-to-noise ratio, due to differences in contrast agent, may be a potential confounder. A prior study has shown good agreement in ventricular volume measurement derived from gadolinium-based contrast enhanced 4D flow and ferumoxytol enhanced 4D flow MRI [24] providing support for comparisons of scans with difference contrast agents. Also, while using the minimum percent of cardiac cycle captured in the voxel-wise tool served to normalize differences in cardiac gating method, this method inherently excludes end-diastole from the hemodynamic measurements. Future studies should limit inclusion to retrospectively gated scans to ensure full cardiac cycle coverage. Additionally, the use of custom software limits the generalizability of these results in a clinical setting. Further, we did not investigate the impact inter-observer variability. A prior study has demonstrated good inter-observer agreement for the caval vein flow distribution measurement [9]. However, the impact of segmentation variability remains to be investigated.

While comparison to routine echocardiography could be potentially helpful, mean and peak blood flow velocities in the caval veins and branch PAs are not systematically reported by echocardiography as they are often inaccurate and limited by acoustic windows. Several studies have established that echocardiography may not identify important anatomic or hemodynamic abnormalities in single ventricle patients, particularly within the extracardiac vasculature [25, 26]. These technical limitations, the physiologic importance of even small pressure gradients in the Fontan pathway, and the potential morbidity of cardiac catheterization have all driven the enthusiasm for cross-sectional imaging, particularly CMR, prior to and following the Fontan procedure [27, 28].

## Conclusions

Longitudinal 4D flow MRI in a cohort of Fontan patients revealed that flow distribution to the pulmonary arteries may be an important driver of worsening flow efficiencies and should be measured in future studies. Our findings also suggest that changes in caval peak velocity, an easy to obtain flow metric measured by MR or echocardiography, may serve as a surrogate for changes flow efficiency quantified by in energy losses. Future, larger longitudinal studies are warranted to validate and further explore these findings.

## Data Availability

The data that support the findings of this study are available on request from the corresponding author EKW. The data are not publicly available due to data sets containing information that could compromise research participant privacy/consent.
